# Why recombination hotspots?

**DOI:** 10.1371/journal.pgen.1012152

**Published:** 2026-05-19

**Authors:** Julien Joseph, Thomas Brazier, Marie Raynaud, Sylvain Glémin, Frédéric Baudat, Bernard de Massy, Nicolas Lartillot, Laurent Duret

**Affiliations:** 1 Université Lyon 1, CNRS, Laboratoire de Biométrie et Biologie Evolutive UMR5558, Villeurbanne, France; 2 Université Lyon 1, CNRS, ENTPE, LEHNA UMR5023, Villeurbanne, France; 3 Université de Rennes, CNRS, ECOBIO, UMR6553, Rennes, France; 4 ISEM, Univ Montpellier, CNRS, IRD, Montpellier, France; 5 Institute of Ecology and Evolution, School of Biological Sciences, University of Edinburgh‌‌, Edinburgh, United Kingdom; 6 IGH, Univ Montpellier, CNRS, Montpellier‌‌, France; University of Washington, UNITED STATES OF AMERICA

## Abstract

Meiotic recombination is the process by which DNA is exchanged between parental chromosomes during the production of gametes in eukaryotes. This phenomenon has important implications for fertility, genetic diversity and genome stability. Intriguingly, not all regions of the genome are equally susceptible to recombine during meiosis. Instead, in many eukaryotes, recombination events are concentrated in short genomic segments called recombination hotspots. Since the first discovery of recombination hotspots, several theories have emerged to explain their existence. In this review, we discuss the relevance of these theories in regards of recent advances in characterizing the diversity and determinants of fine-scale recombination landscapes. Finally, we outline new research avenues for elucidating the evolutionary origins of recombination hotspots.

## 1 Introduction

Meiotic recombination consists in the exchange of genetic information between two homologous chromosomes during the production of gametes. It is initiated by programmed double-strand breaks (DSBs) that locally erase part of the sequence. These breaks can be repaired either by copying the missing DNA from the sister chromatid or by copying the missing DNA from the homologous chromosome (a process called gene conversion). In the latter case, break repair will result in a Crossover (CO) if there is an exchange of chromosome arms on either side of the conversion tract, or in a Non-Crossover (NCO) otherwise. The main hypothesis for the near-ubiquity of meiotic recombination in Eukaryotes is its role in increasing the efficacy of selection by breaking genetic linkage between alleles of different selective values [1–4]. Moreover, in most sexually reproducing species, homologous recombination is crucial for the proper segregation of chromosomes during meiosis and is therefore mandatory for gamete or spore production [5,6]. Nevertheless, despite its obvious importance, recombination also has deleterious consequences. First, it has been shown that recombination can be mutagenic [7–11], and second, that it can induce a fixation bias towards GC alleles through GC-biased gene conversion (gBGC), which can interfere with selection [12–17]. Consequently, a high recombination rate will allow the independent segregation of beneficial and deleterious alleles, but it will potentially increase the mutational load and induce a fixation bias of potentially deleterious GC alleles. It has therefore been proposed that the distribution of recombination events along the genome, the so-called recombination landscape, could evolve to maximize the benefits and minimize the cost of recombination.

Indeed, an intriguing observation is that recombination events are not evenly distributed along the genome (reviewed in [18–20]). First, there is variation at the chromosome scale. As meiosis generally requires at least one CO event per chromosome, shorter chromosomes often experience a higher recombination rate per base pair [5,21–23]. Second, there is variation at the regional scale. For instance, many species show an increase in recombination rate towards the telomeres [22,23] and a suppression in and around centromeres [23,24]. Moreover, due to the coupling between DNA replication and DSB formation [25], recombination rate is also increased in early replicating regions [26–28]. Finally, in many eukaryotes, there is variation at the kilobase scale with some short sequences (~1 kb) experiencing a much higher recombination rate than their flanking sequences [29–31]. These loci are called recombination hotspots.

**Fig 1:**
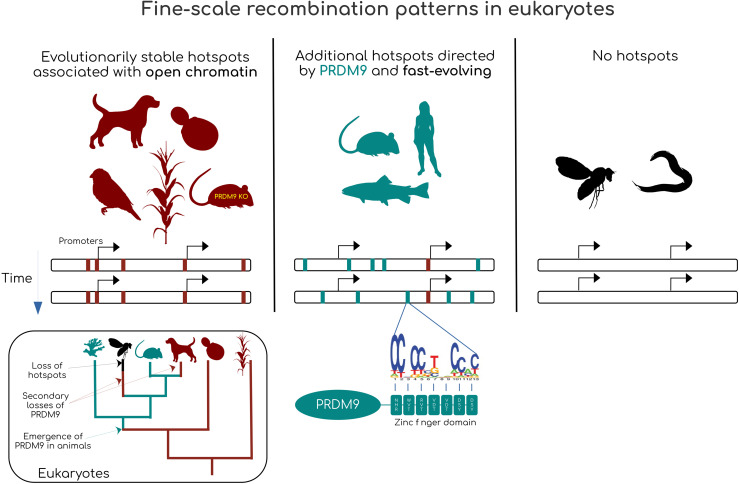
Diversity of the fine-scale recombination landscapes in eukaryotes. Ancestral eukaryotes probably had evolutionarily stable hotspots associated with regions of open chromatin/promoter-like features. In animals, the gene PRDM9 emerged which redirected hotspots outside of promoter-like features. The mode of hotspot specification by PRDM9 induces the erosion of the hotspots. Associated with a high mutation rate of PRDM9’s zinc finger domain, this erosion leads to a rapid turnover of recombination hotspots. Finally, in some animals in which PRDM9 has been lost, promoter-associated hotspots have been lost and recombination does not vary much at a fine-scale. Silhouette images are taken from www.phylopic.org.

Recombination hotspots were first discovered in the fungus *Ascobolus immersus* where a clustering of gene conversion events was observed at the b2 locus [32]. A similar clustering was observed in yeasts, in 5’ of genes such as ARG4 and HIS4 (reviewed in [29]). This observation was later extended to crossovers in many other promoters of protein-coding genes, and these recombination clusters were shown to result from the clustering of programmed DSBs in regions of open chromatin (Fig 1, reviewed in [29] and [33]). Studies in angiosperms and some vertebrates, revealed a very similar pattern, with recombination hotspots often occurring in promoters of protein-coding genes [34–46]. These hotspots appear to be shared by closely related species and are thus relatively evolutionary stable [41,46–50], although some can be species-specific [49,51].

On the other hand, studies in humans, mice, some snakes and some salmonids have revealed another type of recombination hotspots, directed by the protein PRDM9 [46,52–55]. This protein first binds a specific DNA motif with its C2H2 zinc finger array [52–54]. Upon binding, it modifies histones with H3K4Me3 and H3K36Me3 marks [35,56–59]. This signal is recognized by a protein that brings the bound sequence to the chromosomal axis, where it may receive a DSB [58,60–63].

Importantly, the definition of a hotspot is not universal. It depends on the method used to detect recombination (e.g., cytology, linkage disequilibrium (LD), pedigrees) and on the recombination intermediate considered (DSB, CO, NCO). Moreover, it still largely relies on arbitrary thresholds on the length of the sequence considered and on the magnitude of the local recombination elevation. A typical definition for LD-based methods is a 1kb sequence with a recombination rate at least 10 times higher than the regional background [30,64], but alternatives have been proposed [65].

While variation in recombination rate at the chromosomal and regional scale can be linked in part to properties of chromosome organization and chromosome segregation requirements [6,20,28], the “raison d’être” of recombination hotspots remains a mystery.

### 1.1 Recombination hotspots and chromosome pairing

In many species, for a DSB to be repaired and chromosomes to correctly segregate, the nucleoprotein filament that surrounds the single-stranded extremity of the broken DNA needs‌‌ to find its homologous sequence among sometimes billions of base pairs. In this context, focusing the entire process of DSB induction and homology search in a small and pre-defined fraction of the genome appears to be a good way to restrict homology search, and therefore to increase the success of meiosis [66,67]. Recombination hotspots could therefore facilitate homology search and chromosome pairing. Interestingly, despite large-scale variations in recombination rate, some species lack recombination hotspots [24,68]. In several *Drosophila* species, many studies relying on various CO detection methods failed to detect clear hotspots despite sufficient resolution [24,69–73]. Similarly, in *C.elegans*, localized recombination hotpots could not be detected [68,74]. Interestingly, both *Drosophila* and *C.elegans* have a specific coordination of chromosomal events during meiotic prophase, in that synapsis of homologous chromosomes precedes and is independent of recombination [75,76]. It is thus intriguing that the only two species thus far known to lack hotspots are among the few that do not need recombination to pair their chromosomes. This suggests that the concentration of DSBs into hotspots could have evolved to facilitate recombination-dependent chromosome pairing [67]. It would, however, be necessary to statistically assess the association between hotspot presence and recombination-dependent pairing in many other species to properly test this hypothesis.

On the other hand, despite the potential fitness advantage of recombination hotspots for chromosome pairing, it has been shown that the very nature of those hotspots should lead to their erosion through time: the so-called hotspot paradox [77,78].

### 1.2 The hotspot paradox

Several studies reported that in yeasts, when deleting the ARG4 or HIS4 recombination hotspot on one homologous chromosome only, double-stranded breaks (DSBs) systematically occur on the non-deleted copy which is systematically converted by the homologue as a template, and therefore converted into the deleted version [77–81]. This observation revealed what is now called the hotspot paradox: if a hotspot-inactivating mutation occurs within the hotspot, it will systematically convert the functional copy and spread quickly in the population [77,78]. This is a form of biased gene conversion driven by biased formation of the initiating DSB (dBGC). Using simulations, Boulton and colleagues [78] and Coop and Myers [82] showed that this drive was so strong that neither indirect selection on selection efficacy, nor direct selection for correct chromosome pairing was sufficient to counteract it. In summary, in the face of mutations, recombination hotspots should not exist.

Three different processes have or may have helped to maintain hotspots despite the hotspot paradox. The first option would be to target an epigenetic mark whose presence is less dependent on DNA mutations, and more dependent on regional context, as shown in yeasts [29]. Indeed, any mechanism that effectively decouples the locus (or sequence information) that recruits the DSB machinery from the locus where the DSB occurs should resolve the hotspot paradox [83]. A second option would be to target loci under very strong purifying selection for non-meiotic function. This would ensure that new cold alleles are constantly counter-selected. However, this would severely reduce the average fitness of new offspring by recurrently favouring the transmission of deleterious mutations. A third way to overcome the hotspot paradox is illustrated by the PRDM9-dependent mode of hotspot determination. In mice and humans, several studies demonstrated that PRDM9 erodes its targets through the mechanism of dBGC, leading to hotspot extinction [54,60,84,85]. However, mutations on PRDM9 DNA-binding zinc-finger array will often change the binding motifs, and therefore provide new targets [85–87]. As long as the mutation rate of PRDM9’s zinc finger is sufficiently high such that new alleles (and hence new hotspots) appear faster than hotspot erosion, recombination hotspots can persist [67,88–90]. If the mutation rate is not high enough, individuals carrying old PRDM9 alleles will have fewer hotspots and may struggle to complete meiosis, leading to positive selection on new alleles [88,91–93]. Under this dynamic, the zinc finger sequence constantly evolves in order to maintain the same level of fertility. This dynamic has been coined Red-Queen because it is reminiscent of the Red Queen from Lewis Carroll’s Alice’s Adventures in Wonderland: “Now, here, you see, it takes all the running you can do, to keep in the same place.” [94].

Altogether, it is still not clear why some species use recombination hotspots in the first place and why two different hotspot-directing systems coexist, sometimes even within the same species [46,49,85]. In the following sections, we will first discuss the evolutionary processes that could explain the maintenance of promoter-associated hotspots throughout the entire tree of eukaryotes. Second, we discuss the potential reasons behind the evolutionary success of the PRDM9 DSB-directing system in animals. Finally, we provide suggestions to test the hypotheses presented throughout this review.

## 2 The evolutionary origin of PRDM9-independent recombination hotspots

The position of PRDM9-independent hotspots (close to gene promoters) can be explained either by selective pressures that maintain them there, or more simply by an indirect consequence of chromosome organization during prophase I of meiosis. Below, we develop both hypotheses.

### 2.1 Opportunistic recombination hotspots in accessible DNA

The DSB machinery needs access to DNA and has usually little DNA sequence specificity. Therefore, any chromosomal region where DNA is accessible for other reasons (e.g., gene promoter, some regulatory element, replication origin) is a potential recombination hotspot and conversely, any regions with closed chromatin should make recombination more difficult (e.g., centromeres, transcriptionally repressed regions, mating type switching locus).

In yeasts, almost all DSB hotspots are sensitive to DNase I and micrococcal nuclease [95–97]. Therefore, it seems that a primary condition for being a recombination hotspot is nucleosome depletion and open chromatin [29]. This is also the case for maize where open chromatin regions have been associated with recombination hotspots [98,99].

PRDM9-directed hotspots are not necessarily genomic sites of open chromatin, but PRDM9 has the ability to open the chromatin at these sites by modifying chromatin state through histone methylation [35,56–58,100], and to generate a nucleosome-depleted region [101]. It seems very plausible that in the absence of a PRDM9-like system that actively opens chromatin prior to DSB formation, hotspots will naturally occur where the chromatin is already open, which includes, in particular, many transcription start sites.

### 2.2 Selective pressures to maintain the position of PRDM9-independent recombination hotspots

On top of DNA accessibility considerations, there are good reasons to think that the position of PRDM9-independent recombination hotspots close to promoter-like features could provide fitness advantages over flat recombination landscapes. It is therefore possible that the position of PRDM9-independent recombination hotspots in Eukaryotes has been maintained by natural selection.

#### 2.2.1 Selection to maximize Hill-Robertson interference dissipation.

Hill–Robertson interferences arises when alleles of opposite selective values are in genetic linkage [1,2]. Without recombination, the positive selection of the beneficial allele interferes with the elimination of the deleterious one. It has been shown that the recombination landscape that maximizes genetic shuffling (regardless of selective values) is flat [102]. If selected loci were evenly distributed across the genome, this landscape would also maximize Hill–Robertson interference dissipation. However, most of the strongly selected sequences are concentrated in protein-coding genes and are far from being evenly distributed across the genome. Therefore, the position of recombination hotspots in promoters increases genetic shuffling between regions that have strong effects on fitness and should provide an indirect selective advantage over flat landscapes [23,103]. Moreover, it has been suggested that this positioning could participate in decoupling the evolution of a gene from its promoter [103], thereby avoiding potential evolutionary conflicts between genes and their cis-regulatory elements [104]. To test this hypothesis, the selective pressure exerted on a modifier locus affecting the recombination rate between a gene and its promoter could be assessed within the modeling framework of Fyon and Lenormand [105].

#### 2.2.2 Selection to limit ectopic recombination events.

It is worth noting that not all open chromatin regions correspond to recombination hotspots [33]. In dogs and passerines, which both lack PRDM9, only gene promoters that contain a CpG island (regions rich in CpG dinucleotides, generally hypomethylated) are recombination hotspots, rather suggesting a negative association between recombination and DNA methylation [38,40]. Interestingly, nearly all newly hypomethylated regions that appeared in mice became new PRDM9-independent recombination hotspots, specific to the PRDM9-KO mouse [49]. Moreover, in *Ascobolus* fungi, experimentally induced methylation of the b2 hotspot leads to its inactivation [106]. *Arabidopsis thaliana* shows a similar pattern, where experimentally induced DNA methylation is sufficient to silence meiotic recombination [107,108], and conversely, DNA de-methylation activates cross-over activity [109]. Finally, in honey bees, genes that are methylated display a lower rate of cross-overs [110].

Methylation of CpG dinucleotides is a widespread mechanism for transposable element (TE) repression in eukaryotes (reviewed in [111]). In the plant *Arabidopsis thaliana*, but also in the fungus *Neurospora crassa*, methylation of TEs in meiosis is thought to be mediated by an interfering double-stranded RNA which recruits a methyltransferase that methylates CpG dinucleotides [112,113]. In vertebrates, a well-diversified family of C2H2 zinc finger proteins (the KRAB-ZNFs) recognize specific motifs associated with TEs, and eventually recruit a protein that methylates CpG dinucleotides (reviewed in [114]). It has been hypothesized that this methylation decreases the risk of ectopic recombination events by preventing the recombination machinery from accessing repeated elements [106,115,116]. It is also very clear that DNA methylation not only prevents DSBs from occuring in TEs but also represses TE expression and contributes to limiting the transposition-induced mutation load in the germline [117]. Altogether both consequences of DNA methylation are thought to have a positive effect on fitness, and could explain its persistence [106,115,118,119]. Moreover, even if the main driver of selection for TE methylation is the minimization of the mutational load through transcription repression, a recombination machinery that avoids the hallmarks of DNA methylation should, in principle, be selected for, as it should drastically reduce non-allelic pairing and ectopic recombination events [119].

The exact nature of the mechanism that keeps recombination away from methylated regions is still debated, and can differ between species. It has been demonstrated that the histone mark H3K4Me3, which largely colocalizes with DNA hypomethylation, is clearly associated with recombination hotspots [33,35,38]. In yeast, which lack DNA methylation, an inactivation of the H3K4 methylase Set1 leads to a severe reduction of DSB formation at recombination hotspots, which are redistributed across the genome [120,121]. However, a causal role of H3K4Me3 in the positioning of hotspots is much more debated in plants [122,123], where tuning DNA methylation is sufficient to silence or activate meiotic recombination [107–109], without involving H3K4Me3 marks. In vertebrates, it is hard to dissociate the role of H3K4Me3 marks and DNA hypomethylation since they strongly co-localize [124].

If the absence of DNA methylation is the major driver of recombination hotspots in vertebrates rather than H3K4Me3 marks, species that exhibit low or no DNA methylation should recombine everywhere and should therefore largely lack recombination hotspots [116]. Intriguingly, in mice with a DNA methylation-deficient background, the concentration of DMC1 (indicating DSB activity) increases in TEs, outside of canonical, PRDM9-directed recombination hotspots [116], indicating that methylation is a suppressor of recombination activity even in species with PRDM9-dependent hotspots. Mugal and colleagues [125] also showed that bird genomes were much less methylated than those of mammals or fishes. In this context, the whole genome should be more permissive to recombination, and recombination hotspots should be less strong. It is thus interesting to note that passerines show fewer and weaker recombination hotspots than dogs, based on LD maps [38,40,41,126,127]. Finally, in many mammals, the genome is much less methylated in oocytes than in spermatocytes in meiotic prophase I [128]. It is again interesting to note that female recombination in dogs is more evenly distributed and occurs less in canonical recombination hotspots (CpG islands) than in males [126]. All these observations could suggest that the usage of PRDM9-independent recombination hotspots in many vertebrates is a direct consequence of the heterogeneity in methylation levels. A strong testable prediction of this hypothesis is that recombination heterogeneity in PRDM9-lacking species should correlate positively with DNA methylation heterogeneity.

## 3 On the evolutionary advantage of PRDM9-directed recombination hotspots

Full-length PRDM9 homologs (including all necessary domains for its activity in human and mice) have been found in diverse animal clades including Cnidarian and sponges [91,92]. This implies that PRDM9 was present in the ancestor of all animals. Whether PRDM9 kept the same function during the 600 Mys that separate us from this ancestor is still unknown. Evidence from salmonids’ CO and DSB maps clearly demonstrates that PRDM9 is also involved in determining the position of recombination hotspots in this family [55]. Its function can therefore be traced back at least to the ancestor of Euteleostomi (~420 Mya) [129].

The inactivation of PRDM9 by KO experiments in male mice and rats leads to a drastic decrease in fertility due to an inability to efficiently repair DSBs generated at the “default” location of hotspots (in CpG islands) [35,130–132]. Both its persistence through time and lineages, and the direct evidence in murids suggest that PRDM9 is under strong selective constraints and should thus provide a critical fitness advantage. In this section, we will discuss several hypotheses that have been formulated for the evolutionary advantage of PRDM9 in regards of the recent literature on model and non-model species.

### 3.1 Selection to limit the deleterious effects of recombination in functional sequences

Even before the role of PRDM9 in meiosis was understood, it was noticed that in humans and mice, unlike in plants and fungi, recombination hotspots were not associated with promoter-like features [29,30]. In regard of the damage that recombination can induce via its mutagenic effects or via biased gene conversion, it has been naturally formulated that one evolutionary advantage of PRDM9 could be to deviate recombination away from these promoter-like features, hence reducing the genome-wide genetic load [35,133]. In this sense, it has been demonstrated that COs were depleted in the body of meiotically transcribed genes in both humans and mice [134–138]. It has been further proposed that the depletion of PRDM9-directed COs in the body of genes could be an advantage to reduce interference between the transcription and the recombination machineries which both need access to DNA in meiosis [136,138].

**Fig 2:**
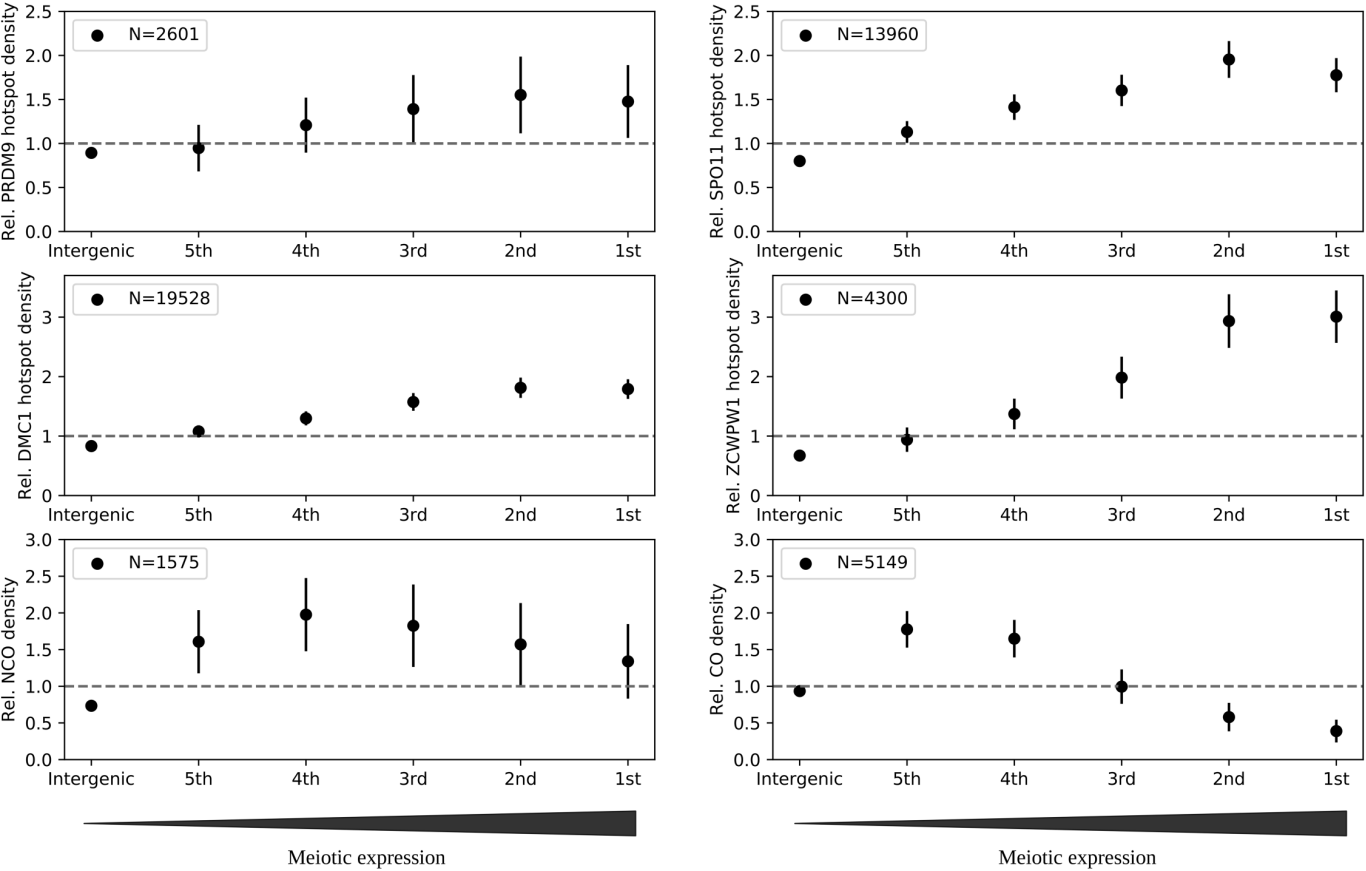
Relative hotspot density as a function of meiotic expression in mice. The density of PRDM9 hotspots in mice was measured with the maps of PRDM9 hotspots constructed by Chip-seq experiment on male mice in [59]. The SPO11 hotspot density was obtained from SPO11 oligo-seq performed by [139]. The ZCWPW1 hotspots was obtained from the CUT&RUN experiments of [140]. DMC1 hotspots in B6 mice were retrieved from the study of [85]. The CO and NCO events come from the study of [61] and [62]. They were obtained by sperm sequencing of B6/CAST hybrid mice. We considered six categories of transcription activity: five quantiles of gene expression in leptotene retrieved from the study of [141], and intergenic regions. Hotspot density was computed as the number of peaks/events that overlapped each category, divided by the sum of the length of the transcripts of this category. This density was normalized by the total number of peaks/events called in the whole genome. For all the overlaps, we excluded blacklisted regions in which peaks cannot be called. Error bars correspond to binomial error in the sampling of hotspots. Note that NCO density corresponds to observed NCO event whose detection power depends on heterozygocity which can vary between gene expression categories.

**Fig 3:**
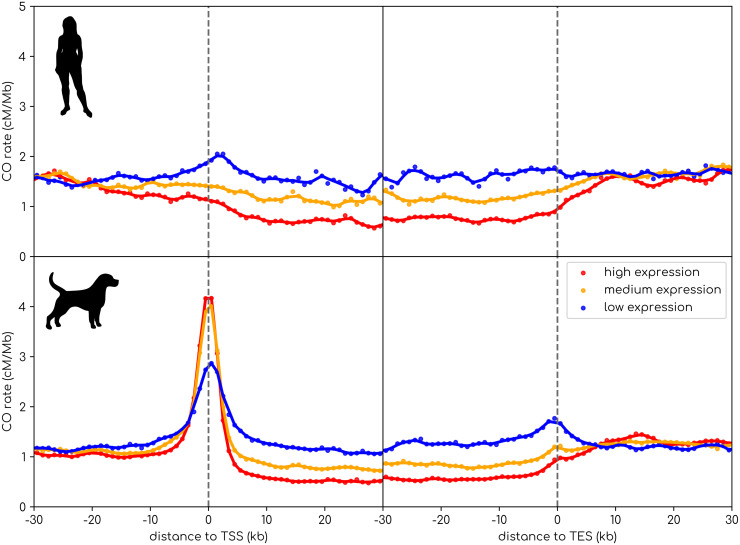
Deficit of CO rate in the body of highly expressed genes in dogs and humans. We cut by bins of distance to the nearest TSS or TES. All dot between the TSS and TES are inside a protein-coding gene. We distinguished 3 equal sized categories of genes regarding their expression in testis for dog [150] and in ovary for humans [151,152]. We kept only genes that have detectable expression in dog testis or human ovary (Dog: *N* = 23,116 genes, Humans: *N* = 20,050 genes). The sex-averaged recombination rates in humans were estimated from large pedigrees in [153]. The dog recombination map was estimated from linkage disequilibrium in [38]. Silhouette images are taken from www.phylopic.org.

In principle, the depletion of COs in genes could be due either to a lower DSB activity or to a bias in DSB repair towards NCOs. Here, using markers of the different steps of the recombination pathway in a B6 mouse strains, we confirm that while the CO rate is lower in highly meiotically expressed genes, the DSB rate is in fact higher [10,11,58,137] (Fig 2). This suggests that the crossover deficit does not result from an avoidance of PRDM9 binding in meiotically expressed genes but from how DSBs are repaired in those genes. Importantly, this shows that these genes still suffer the cost of DSB-induced mutagenesis and NCO gene conversion. Moreover, we can also observe that in dogs, which have lost PRDM9, there is also a deficit in COs in highly expressed genes (Fig 3). Altogether, we show that PRDM9 cannot be held responsible for the depletion of COs in the body of meiotically expressed genes. Still, it is possible that PRDM9 reduces the genetic load in hypomethylated promoters of highly expressed genes (Fig 3). Below, we discuss the robustness of this hypothesis in regard of the strength of the mutagenic effect of recombination and the load incurred by gBGC.

#### 3.1.1 The mutagenic effect of recombination.

Several studies showed that DSBs and COs are associated with an increase in mutation rates in humans and yeasts [7–11,142–144]. In humans, a recent estimation indicates that a DSB can result in up to a 400-fold increase in the mutation rate in the broken region [10]. However, given that the probability of having a DSB in a given recombination hotspot in a given meiosis is very low, this only leads to a 40% increase of the mutation rate in the strongest PRDM9-directed hotspots [10]. It has been reported in dogs, birds and a PRDM9 KO mouse that recombination hotspots appear to be less strong than PRDM9-directed ones [35,38,40,126,127]. One can therefore expect that the increase in mutation rate will be less important in those hotspots. It is therefore not clear whether the recombination-induced mutation rate in promoter-associated hotspots can lead to sufficiently strong indirect selection for an alternative system. It would be interesting to test this point with formal population genetics models.

#### 3.1.2 Is gBGC deleterious?.

As explained in the introduction, gBGC can be another source of recombination-induced genetic load [15,145]. In this direction, empirical studies showed that gBGC recurrently leads to the fixation of slightly deleterious GC-alleles in recombination hotspots [14,16,17,146,147]. However, a recent theoretical study demonstrated that this gBGC load of slightly deleterious mutations does not imply that gBGC is negatively selected [148]. Indeed, this load must be balanced with the individual benefits of purging strongly deleterious recent mutations [145], predominantly toward AT [149], which is difficult to quantify empirically [148]. In this light, it is possible that levels of gBGC observed in functionally important sequences in PRDM9-lacking species in fact improves individual fitness on average, which would favour the preservation of PRDM9-independent recombination hotspots. Consequently, the role of gBGC in the evolution of recombination landscapes remains unclear, and would require further investigation [148].

#### 3.1.3 PRDM9 and transposable elements.

**Fig 4:**
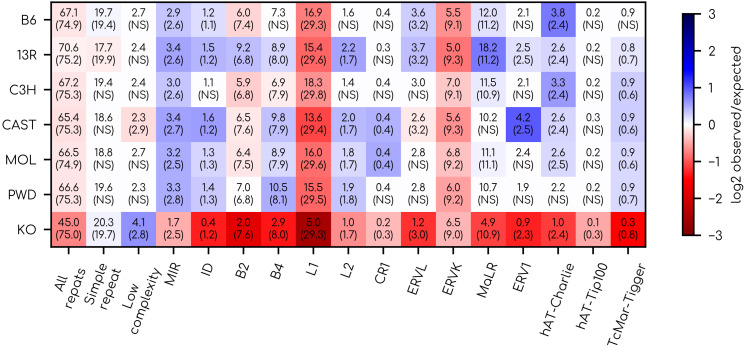
Distribution of mice DSB hotspots relative to repeated elements. We retrieved hotspots of DMC1-ChiP-seq signal from the study of [85]. Random expectation was computed as the mean of the overlap of repeated elements and 40 sets of random DMC1 ChiP-seq peaks (see methods). If the value of the true overlap lies between those of the 40 sets of random DMC1 ChiP-seq peaks, the enrichment is not significant (NS). For a given allele, numbers correspond to the percentage of all DMC1 ChiP-seq peaks whose center is at less than 200 bp from a given repeat family. Numbers in parentheses correspond to the random expectation of the percentage of overlap.

While PRDM9-independent hotspots might increase the genetic load in selectively constrained hypomethylated regions, they should, in principle, avoid transposable elements which are often methylated. Therefore, they should at least provide a way to avoid ectopic recombination events in repeated elements, which can affect large portions of the genome. Because PRDM9 targets short, specific DNA motifs throughout the entire genome, it cannot offer this guarantee. On the contrary, in humans, there is an enrichment of CO hotspots in some transposable elements, particularly in Alu repeats [30]. Buard and colleagues [86] showed that the predicted binding motif of many mouse PRDM9 alleles showed an enrichment in transposable elements, and Yamada and colleagues [154] showed that SPO11 hotspots from the mouse B6 strain allele were enriched in several TE families. Here, using DMC1 ChIP-seq data, we show that the mice DSB hotspots of 6 different PRDM9 alleles are also enriched near several TE families, and we confirm that PRDM9-independent DSB hotspots are clearly depleted in TEs (Fig 4).

We computed the overlap of DMC1 chip-seq peaks with repeated elements for each of the six alleles of the study of [85]. We then randomised the position of the DSB hotspots to estimate whether they were more likely to occur near some TE families than by chance. Importantly, not all TE families are equally susceptible to introduce ectopic recombination events. Long repeats such as L1 and ERV have a higher risk of causing ectopic recombination events than repeats that are small compared to the resection tract length [10].

We can see that DSB hotspots of all alleles are depleted around L1 and ERVK TEs (Fig 4). This could reflect the fact that those TEs are in regions difficult to access for the DSB machinery. On the other hand, the DSB hotspots of all alleles are enriched in the MIR family. Interestingly, we can see that the DSB hotpots of 13R are enriched in 10 out of 16 families of TEs, and 4–8 out of 16 for other alleles (Fig 4). It is particularly enriched in the MaLR family which are responsible for around 1,200 additional DSB hotspots for 13R. We can also see that the DSB hotspots of CAST are strongly enriched in the ERV1 TE family (twice more than by chance) (Fig 4). In bright contrast, PRDM9-independent DSB hotspots (KO) are clearly depleted in all repeat families except for low complexity repeats for which there is an enrichment (Fig 4). Overall, PRDM9 alleles have an additional 20% to 25% of their targets around repeated elements compared to the “default” system (Fig 4). Pratto and colleagues [8] showed that half of the disease-associated non-allelic homologous structural variants reported in humans occur in the DSB hotspots of the PRDM9*^A^* allele, and that most of them occur within low copy repeats. This represents direct evidence that PRDM9-directed hotspots increase the load when targeting repeated elements [155]. The fitness advantage of deviating DSBs away from single-copy promoter-like features is therefore not very obvious.

One other major shortcoming of PRDM9-directed hotspots is their short lifespan. Indeed, because of dBGC, a new PRDM9 allele progressively loses its target, potentially negatively impacting the fertility of its bearers. It has thus been proposed that targeting actively duplicating elements could be a way for an allele to never run short of targets [154]. Additionally, eroding active transposable elements could provide a way to control their expansion [154]. However, it is not clear how those weak indirect advantages scale with the direct deleterious effect of ectopic recombination events in repeated sequences [118,119].

### 3.2 The advantage of coupling DSB formation and repair

As previously explained, the main advantage of recombination hotspots could be to facilitate recombination-dependent chromosome pairing by restricting the homology search space to short genomic regions. One way to achieve this is to limit both DSB induction and homology search to sequences in spatial proximity to the chromosomal axis [66]. In mice, PRDM9-bound sites are brought back to the chromosomal axis [63,137], with the help of MEI4 and IHO1 [63]. Importantly, PRDM9 often binds its target symmetrically on both homologs [60–62,85]. By doing so, PRDM9 may relocate both the sequence that will receive the DSB and the homologous sequence that will be used as a template for its repair in the vicinity of the chromosomal axis. PRDM9 would therefore confer a strong advantage in coupling DSB formation to its repair [58,61,62,66,156]. A strong binding of PRDM9 is key for this process, as the probability of symmetrical binding directly depends on the affinity of the DSB-directing protein for its target, as well as its concentration in the cell [67,89]. In yeast, the PHD finger module of Spp1 is able to read H3K4Me3 epigenetic marks, and tethers the DNA to a protein complex located to the chromosomal axis (through Spp1 interaction with Mer2, the yeast ortholog of mouse IHO1), where it will receive a DSB. However, it is currently unknown to which extant Spp1 binds its target symmetrically and whether bringing both homologs to the DSB site also facilitates DSB repair at PRDM9-independent hotspots in general. A possibility would be that PRDM9 has been selected only because of a higher affinity for its targets. In this view, PRDM9 might have acted as a booster of the DSB repair efficiency, allowing faster meiosis, with potentially fewer resources invested in DSB formation [62,67,89,132,131].

**Fig 5:**
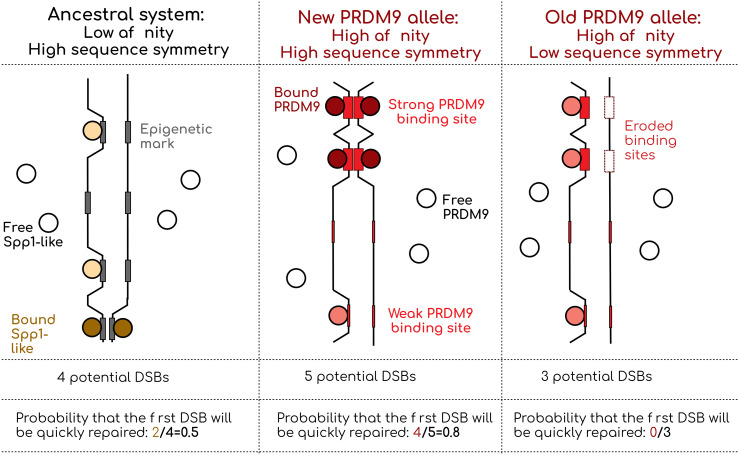
Model of the role of the default DSB-directing system (Spp1-like) and PRDM9 in the formation of crossovers. In this schematic example, the binding probability of the DSB inducer is 0.5 for epigenetic marks, 0.25 for weak PRDM9 binding sites and 1 for strong PRDM9 binding sites. In this arbitrary setting, before the erosion of strong binding sites, PRDM9 provides an advantage compared to the default system to efficiently repair DSBs. After the erosion of strong binding sites, the default system becomes more advantageous. One can hint from this schematic representation that the distribution of binding probability of potential DSB targets, as well as the erosion level of high-affinity PRDM9 targets will be key to determine which system is more efficient.

On the other hand, it is interesting to note that after 600 Mys, the “default” system has not disappeared and still plays a significant role in determining recombination hotspots in species with a fully functional PRDM9 [43,46,49,85]. If this default system is completely opportunistic and does not require any additional steps other than those of the PRDM9 system, its persistence could simply be passive. However, it is also possible that the default system has been maintained by natural selection. Indeed, the decrease in fitness of old alleles that fuels PRDM9’s Red Queen dynamic could also maintain an alternative mechanism: in any individual who should struggle to make enough CO because of an old, inefficient PRDM9 allele, a mutation that increases the efficiency of the default system can be positively selected (Fig 5). This verbal model should be tested theoretically, for example, within the modeling framework of [67] or [89].

Of note, there are sex differences in PRDM9-independent hotspot usage in species with PRDM9. In these species, this difference may be linked to sex differences in the efficiency of the PRDM9 system and not so much with gametocyte DNA methylation (see section 2.2.2). In mice, the usage of PRDM9-independent hotspot usage strongly depends on the PRDM9 allele in males [85,131], and varies between females, even with the same PRDM9 allele [157]. Understanding sex differences in PRDM9-independent hotspot usage in a wider range of species with and without PRDM9 should shed more light on the constraints exerted on the usage of these hotspots.

Overall, it is possible that PRDM9 has been selected for as a DSB repair facilitator, and not so much for deviating recombination away from promoter-like features. The fluctuating position of PRDM9-directed hotspots may therefore be a byproduct of this system, rather than a selected trait conferring an independent fitness advantage. However, in theory, a “non-paradoxical” system could reach the same affinity for its targets as PRDM9, without triggering dBGC. With this system, DSB repair would reach the same efficiency, without the cost of hotspot erosion, and should therefore provide higher fertility on average. To resolve this question, it is crucial to investigate whether/how DSB formation is coupled to repair in PRDM9-lacking species.

## 4 Conclusions and future directions

**Fig 6:**
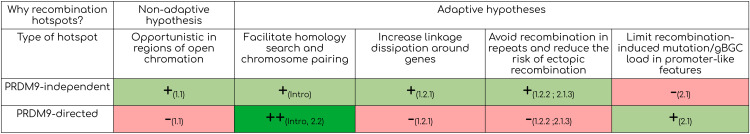
Summary of the different hypotheses for the evolutionary origins of the two types of hotspots presented throughout this review, along with the sections of the manuscript discussing them.

The location of hotspots near gene promoters could simply be the result of opportunistic behaviour of the recombination machinery, which needs open chromatin to access DNA [29]. In this hypothesis, the positioning of hotspots is unrelated to the costs and benefits of recombination (Fig 6). Alternatively, the strong association between PRDM9-independent recombination hotspots and hypomethylation could suggest that selection to avoid repeated elements may have played a role in their maintenance [110,122,158]. The observation of many disease-associated ectopic recombination events in low-copy repeats in humans supports this hypothesis [8]. However, whether ectopic recombination events are less frequent in PRDM9-lacking species remains to be demonstrated.

Overall, the very existence of promoter-associated hotspots may just be a coincidental consequence of a combination of specific requirements such as chromatin state and occupancy, or avoidance of repeated elements (Fig 6). These requirements may happen to be jointly met only for a tiny fraction of the genome strongly enriched in gene promoters.

As PRDM9’s first discovered role was to determine the position of recombination hotspots mainly outside of promoter-like features, it has been hypothesized that it could reduce the recombination-induced genetic load [35,133,159]. Here, we first confirmed that PRDM9 does not deviate DSBs away from gene body, and that the deficit of crossover in meiotically expressed genes is not restricted to species with PRDM9. In addition, it seems unclear whether the load induced by recombining within hypomethylated regions is higher than that of recombining outside, risking an increase in ectopic recombination events [8]. To answer this question, there is a need for theoretical and empirical studies that can compare the burden of recombination-induced mutagenic effects in functional sequences with the fitness costs of ectopic recombination (Fig 6).

Alternatively, the main advantage of PRDM9 could be to promote efficient coupling between DSB formation and repair, owing to the high affinity of PRDM9 for its targets [62,67]. To test this hypothesis, we need to understand whether and how repair is coupled to DSB formation in PRDM9-independent systems and compare their efficiency to the PRDM9 system, for instance in term of DSB required per crossover.

Finally, some species do have recombination hotspots, but their localization significantly departs from the two main patterns discussed in this review. For instance, in the common aspen (*Populus tremula*), recombination hotspots seem to be concentrated in transcription ending sites [45], a pattern which still need to be explained both from a mechanistic and evolutionary perspective. This highlights the importance to further investigate the mechanisms of hotspot regulation in diverse species, enabling comparative studies across species with various recombination landscapes.

One complexity in such comparative studies can be the lack of a harmonized definition of what is a recombination hotspot mentioned earlier in the manuscript. To date, hotspots have been broadly defined as small regions with an above-average frequency of recombination events. However, this definition - in terms of hotspot size and intensity, and therefore their ease of detection, depends on the method of measurement (e.g., sperm typing, ChIP-seq, pedigree or population-based maps (reviewed in [20,39,160]) and on the study. Developing harmonized measures of recombination hotspot location and usage for different detection methods, as well as complementary descriptors of fine-scale recombination heterogeneity, would improve comparability between studies and deepen our understanding of the evolution of hotspots.

## 5 Materials and methods

### 5.1 Expression datasets and recombination maps

Expression in leptotene was obtained from single-cell experiment in mice carrying a B6 PRDM9 allele from [141]. The expression data in humans were obtained from the RNA-seq experiment on human ovaries performed by [151,152] and extracted from the supplementary data of [136]. Expression in ovaries are the ones that show strongest correlation with recombination [135,136] and are therefore considered to be the closest to a meiotic expression profile. The dog expression data is from the RNA-seq experiment of Chen and colleagues (2018) on male testis [150] (GEO accession number GSE106077). The expression were not measured particularly on meiotic cells, but it is the closest transcription profile to a meiotic one which is available on GEO for dogs. The sex-averaged recombination rates in humans were estimated from large pedigrees in [153]. The dog recombination map was estimated from linkage disequilibrium in [38].

### 5.2 Mouse hotspot, CO and NCO datasets

The density of PRDM9 hotspots in mice was measured with the maps of PRDM9 hotspots constructed by Chip-seq experiment on male mice in [59] (GEO accession number GSE93955). The SPO11 hotspot density was obtained from SPO11 oligo-seq performed by [139]. The ZCWPW1 hotspots were obtained from the CUT&RUN experiments of [140] (GEO accession number GSE139289). SPO11, PRDM9 and ZCWPW1 hotspots used in this study come from mice carrying the B6 allele of PRDM9. DSB hotspots were retrieved from the study of [85] (GEO accession number GSE75419). The dataset contains the positions of hotspots called on DMC1 Chip-seq experiments in meiotic cells on seven individuals, six homozygous for a given PRDM9 allele (B6, CAST, 13R, C3H, MOL and PWD), and one whose PRDM9 has been inactivated with a knock-out (KO) (GEO accession number GSE75419). The CO and NCO events come from the study of [61] and [62]. They were obtained by sperm sequencing of B6/CAST hybrid mice.

### 5.3 Enrichment in repeated elements

The positions of repeated elements on the mm10 reference genome were downloaded from the UCSC Table Browser. We considered in the analyses the 15 most abundant TE classes along with simple and low complexity repeats. For each allele, we computed the overlap between each family of repeated elements and the center of hotspots ±100 bp. We then created 40 instances of control sequences by shuffling the hotspots in the genome. If a region of the genome of more than kb had zero SSDS reads in the ChIP-seq reads, this region was excluded from the potential random hotspots because it is likely that hotspots would not be callable even if they existed. We also excluded the ChIP-seq blacklisted regions from the ENCODE project [161], which had been excluded in the original study because it resulted in spurious ChIP-seq peaks. The overlap expected by chance was taken as the mean of the overlap between the 40 sets of random hotspots and repeated elements. If the true value of the overlap was within the values of the 40 sets of random hotspots, the enrichment was not considered as significant.

**Key learning points**

- Recombination hotspots are widespread in eukaryotes.
- Two types have been described so far: one type depends on the PRDM9 protein, and the other type occurs in open chromatin regions.
- Hotspots may have evolved because they promote efficient chromosome pairing, especially PRDM9-dependent hotspots.
- These two types of hotspots have different effects on the mutation load and genome stability.
- Further studies are needed to characterize the diversity of recombination hotspots and their evolutionary consequences.

**Top 5 papers**

- Boulton, Alan, Richard S. Myers, and Rosemary J. Redfield. 1997. “The Hotspot Conversion Paradox and the Evolution of Meiotic Recombination.” Proceedings of the National Academy of Sciences 94 (15): 8058–63. https://doi.org/10.1073/pnas.94.15.8058.
- Brick, Kevin, Fatima Smagulova, Pavel Khil, R. Daniel Camerini-Otero, and Galina V. Petukhova. 2012. “Genetic Recombination Is Directed Away from Functional Genomic Elements in Mice.” Nature 485 (7400): 642–45. https://doi.org/10.1038/nature11089.
- He, Yan, Minghui Wang, Stefanie Dukowic-Schulze, et al. 2017. “Genomic Features Shaping the Landscape of Meiotic Double-Strand-Break Hotspots in Maize.” Proceedings of the National Academy of Sciences 114 (46): 12231–36. https://doi.org/10.1073/pnas.1713225114.
- Tock, Andrew J., and Ian R. Henderson. 2018. “Hotspots for Initiation of Meiotic Recombination.” Frontiers in Genetics 9 (November): 521. https://doi.org/10.3389/fgene.2018.00521.
- Baker, Zachary, Molly Przeworski, and Guy Sella. 2023. “Down the Penrose Stairs, or How Selection for Fewer Recombination Hotspots Maintains Their Existence.” eLife 12 (October): e83769. https://doi.org/10.7554/eLife.83769.
